# Electronic distance based clinical skills training development in family medicine in Namibia

**DOI:** 10.4102/phcfm.v16i1.4352

**Published:** 2024-05-09

**Authors:** Zelra Malan, Felicia Christians, Jan Kuehne

**Affiliations:** 1 Department of Medical Sciences, Faculty of Health Sciences & Veterinary Medicine, University of Namibia, Windhoek, Namibia

**Keywords:** innovation, education, family medicine, clinical teaching, community based education

## Abstract

Workplace-based evaluation is one of the most important, but challenging aspects of medical education. The aim was to improve the assessment of the rural community-based clinical training for undergraduate 3rd and 4th year family medicine students at the University of Namibia (UNAM) and implement a paperless process. An online module was developed on the Moodle platform to include a study guide, an electronic portfolio, and electronic resources (e-books and apps) to replace the current paper version of the logbook. We explored local resources by engaging with students and clinical trainers on how to best conduct the initial implementation. Engagement also entailed motivating students to actively participate in the implementation process. All 3rd and 4th year community-based education end service (COBES) students are now submitting proof of clinical learning electronically with the use of their phones in their online portfolio and using online resources. In addition, students in the practical family medicine module that has been introduced in the 6th year since 2023 are now also using an electronic portfolio and these assessment tools.

**Contribution:**

Overall feedback from students and supervisors indicates a positive atmosphere of learning and constructive feedback on performance from all team members, hopefully improving work-based assessments and ultimately patient care. More members of the primary health care team were involved and the carbon footprint has also been decreased.

## Background

Namibia is one of the least densely populated countries in Africa. The health services consist of two sectors: private (serving 18% of the population with medical aid) and public (serving the remaining 82%). Access to healthcare is comparably good with 76% of the population living within a 10 km radius of a healthcare facility. However, Namibia faces many challenges related to the provision of patient-centred primary health care.^1^ The University of Namibia (UNAM) School of Medicine was established in 2010 and undergraduate family medicine training was first introduced in 2016.

The community-based education end service module (COBES) training requires the 3rd and 4th year medical students consequently to live and work in a rural facility and community. The focus of the placement is the practical implementation of clinical skills and knowledge in a primary care context. A typical class would consist of 70–80 students placed in about 20 small to medium-sized regional and district level hospitals in the rural areas of Namibia for a 4-week placement. In Namibia, most of the rural areas have a hospital that functions as the first point of contact for most patients, for example, historical missionary hospitals. Students are expected to self-select in small groups to designated settings.

Workplace-based learning is one of the most important, but challenging aspects of medical education.^2^ Initially, students were assessed by means of a paper-based logbook, which required only signatures from supervisors to prove successful completion of skills with no feedback on performance. These logbooks and signatures were then assessed by the module coordinator.

We decided that we wanted to improve our assessment for this clinical training. Although workplace-based assessments are widely recognised as valuable tools for evaluating the competency and performance of medical students we were faced with several considerations to ensure effectiveness and feasibility in our setting.^2^ Remote facilities are often faced with limited resources and infrastructure, such as limited supervisors, limited internet access, and language and cultural barriers. We therefore supplied students with data vouchers to enable them to access the online module, upload photos of the tools and assignments, and utilise online resources remotely from their phones. We wanted to be innovative in trying to adapt assessment methods to the available situation while still maintaining standards.

## Objectives

The overall aim was to improve patient care by improving the quality of educational assessment and specifically feedback in the clinical setting, as well as the following:

- to improve direct observation and feedback of medical students who deal with actual patients in a real workplace for increased performance-based assessment
- to improve constructive feedback on performance
- to involve more members of the primary health care team in assessment
- to reduce carbon footprint and move to an electronic paperless system.

## Methods

Firstly, we developed the online module on the Moodle platform to include a study guide, an electronic portfolio, and electronic resources (e-books and apps) to replace the paper version of the logbook. A list of examples of clinical skills performed in a primary setting is included in the study guide. The portfolio includes a section on the assessment tools, all assignments, and student feedback on the experience. We introduced 3rd and 4th year students in smaller groups to the online module and practical and technical aspects of using it during hands-on sessions.

To improve direct observation in the clinical workplace, we introduced the students to the mini-Clinical Evaluation Exercise (mini-CEX) and the Direct Observation of Procedural Skills (DOPS), before they left for the health facilities.^3,4^ We shared an online one-pager of how to use the tools, which are included in the online study guide and asked them to peer evaluate each other using the tool in a practical session. Each student received a one-pager with four versions of each tool, printed double-sided, kept by them, and used for assessment. This entails that each student received three pages when they started the rotation. Students were then asked to take a photograph of the completed page (with four tools) and upload it on a weekly basis electronically to the module.

Secondly, we championed a group leader from each student group and asked them to facilitate a student presentation to the facility manager and other members of the primary care team. Consequently, we engaged with the different healthcare facility managers, informing them of the new assessment methods, and that students will be meeting with them and presenting the tools at the start of the rotation.

To involve more members of the primary health care team, we informed students that assessors can be anyone with expertise in the procedure or consultation, including nurses, doctors, and allied health professionals, as appropriate. Not all elements need to be assessed on each occasion and need to be tailor-made to the opportunities available at the specific facility. Students who completed the Brief Behaviour Change Counselling (BBCC) online training were asked to peer-review each other with the validated ABC tool.^5^

Lastly, we realised that internet and/or cell phone coverage is not widely available. The cost saving for not having to print logbooks enabled us to provide each student with a N$ 100.00 (Namibian dollar) data voucher. This allowed students to upload pictures and access the module from their phones at the training site.

## Results

All 3rd and 4th year COBES students are now being assessed with these tools, which they submit electronically in their online portfolio, as proof of their clinical learning. Students upload photos when they are at the site on a weekly basis. A minimum of four DOPS and two Mini CEX tools are required per week to enable reflection on performance and to identify gaps in clinical skills. Academic supervisors at UNAM check each student’s weekly progress online and communicate with them regarding their performance on a weekly basis. Furthermore, students in the practical family medicine module that has been introduced in the 6th year since 2023 are now also using an electronic portfolio and assessment tools.

More members of the primary health care teams are involved in assessment and students report that nurses specifically, are extremely keen on using the DOPS tool to assess and give feedback.

Overall feedback from students and supervisors indicates a positive atmosphere of learning and constructive feedback on performance from all team members, hopefully improving work-based assessments and ultimately patient care.
